# Enhanced Topical Delivery of Methotrexate via Transferosome-Loaded Microneedle Array Patch: Formulation, Optimization, and In Vitro–In Vivo Assessment

**DOI:** 10.3390/ph18040594

**Published:** 2025-04-18

**Authors:** Snehal Shinde, Anil Kumar Singh, Vijay R. Chidrawar, Amarjitsing Rajput, Sudarshan Singh

**Affiliations:** 1 Department of Pharmaceutics, Poona College of Pharmacy, Bharati Vidyapeeth Deemed to be University, Erandwane, Pune 411038, Maharashtra, India; snehalshinde1004@gmail.com; 2 United Institute of Pharmacy, Prayagraj 211010, Uttar Pradesh, India; singhanil2682@gmail.com; 3 School of Pharmacy and Technology Management, SVKM’s Narsee Monjee Institute of Management Studies (NMIMS), Deemed-to-University, Green Industrial Park, TSIIC, Mahbubnagar 509301, Telangana, India; vijay.chidrawar@gmail.com; 4 Faculty of Pharmacy, Chiang Mai University, Chiang Mai 50200, Thailand; 5 Office of Research Administration, Chiang Mai University, Chiang Mai 50200, Thailand

**Keywords:** design of experiment, dissolving microneedle, methotrexate, microneedle array patch, transferosomes

## Abstract

**Background:** Conventional approaches in treating psoriasis demonstrate several complications. methotrexate (MTX) has been frequently used for its efficacy in managing moderate to severe psoriasis. However, MTX acts as an antagonist in regular dosage, which creates a patient compliance issue with undesirable consequences for patients, which necessitates development of an innovative approach to enhance skin permeation. Therefore, this study examines the improved topical administration of MTX utilizing a transferosome-loaded microneedle (MNs) array patch for the management of psoriasis. **Methods:** A design of experiment was used assess the effect of phospholipid content and edge activator type on vesicle size and entrapment efficiency (EE) to fabricate and optimize transferosome-loaded MTX. Furthermore, the MTX was incorporated within MNs and assessed for in vitro-ex vivo-in vivo parameters. **Results:** The morphology result revealed vesicles mean diameter of 169.4 ± 0.40 nm and EE of 69 ± 0.48 (%). Compared to traditional formulations (MTX patch and gel), the optimized transferosome-loaded dissolving MN array patch showed a substantial increase in diffusion of MTX tested over rat skin. Furthermore, an enhanced therapeutic benefit at the application site through cumulative drug release profiles suggested sustained release of MTX over 24 h. Moreover, in vivo experiments showed that the MN array patch exhibited higher accumulation, compared to conventional formulation tested. In addition, the plasma concentration measurements demonstrated a reduction in systemic exposure to MTX, diminishing the possibility of intricacy while preserving localized therapeutic efficacy. The capability of the MN array patch to lance the epidermal layers was proven by histological assessments. **Conclusions:** Thus, transferosome-loaded MNs is a viable method of delivering MTX topically with prolonged drug release and reduced systemic toxicity.

## 1. Introduction

The skin, as the body’s largest organ, plays a crucial role in protecting against environmental factors and maintaining homeostasis. However, it is also susceptible to various dermal complications that can arise from both intrinsic and extrinsic factors. Psoriasis has a long and intricate history that goes back thousands of years. Ancient books from many different civilizations have allusions to skin disorders that resemble psoriasis. The Greek physician Galen’s origin termed “psoriasis” refers to a scaly, itchy skin ailment [1]. However, psoriasis was not identified as a separate illness from other skin conditions like leprosy until the 19th century. Understanding psoriasis has changed dramatically from a stigmatized and recognized chronic autoimmune disease affecting 2% of the world’s population [2]. Psoriasis has a complicated pathophysiology influenced by immunological, environmental, and other factors. Thick, scaly plaques grow on the skin due to an inflammatory response of lymphocytes [3]. Although psoriasis mainly affects the skin, it is also linked to systemic comorbidities, mainly psoriatic arthritis, cardiovascular disorders, and mental health problems, which make managing it more difficult and negatively impair the quality of life for those who suffer from it [4]. Recent developments in psoriasis treatment have greatly expanded through available alternatives, especially with the advent of new biological agents and creative delivery methods. Notably, the FDA approved bimekizumab, an interleukin-17 A and F blocker, in October 2023 after it showed significant effectiveness in treating passable to acute plaque psoriasis. In addition to improving therapeutic outcomes, this dual-target strategy has a better safety profile than conventional treatment [5]. A recent clinical trial study on rhodomyrtone-fortified vesicles demonstrated significant improvement in the management of psoriasis lesions [6].

Conventional approaches in treating psoriasis involve topical therapy, phototherapy, and systemic use of drug-like MTX. Because of its anti-inflammatory and immunosuppressive qualities, MTX has been frequently used for its efficacy in managing moderate to severe psoriasis. However, MTX acts as an antagonist in regular dosage, which creates a patient compliance issue with undesirable consequences for patients [7]. Moreover, its low aqueous solubility (<1 mg/mL), suboptimal lipophilicity (log P −1.8), high molecular weight (454.4 Da), and ionization at physiological skin pH (~5.5 limiting passive diffusion) the transdermal delivery faces challenges due to a “double barrier”, where it fails to dissolve effectively in aqueous formulations or partition into the skin’s lipid-rich stratum corneum. This necessitates innovative approaches to enhance skin permeation [8]. Thus, to maximize complex MTX administration for psoriatic lesions. Increased interest is in developing novel drug delivery systems while reducing systemic exposure and adverse effects. One possible strategy is using the ability to penetrate the stratum corneum, the skin’s outermost layer. It has been demonstrated that several compounds, including MTX, possess the ability to puncture and deposit into the skin effectively through a technology-based delivering approach [9].

Transferosomes address these challenges via ultra-deformable vesicles (deformability index 27.13) composed of phospholipids and edge activators (e.g., surfactants), which squeeze through tight intercellular spaces in the stratum corneum and enhance drug solubility. These vesicles increase transdermal flux by ~3× (28.12 vs. 10.35 µg/cm²/h in conventional gels) [10] by bypassing the skin barrier and enabling sustained release, while their lipid bilayer structure improves compatibility with both low aqueous soluble (ionized MTX) and lipophilic skin environments [11]. Recent advancements in drug delivery technologies have focused on improving the localized application of MTX through methods such as nanoparticles, vesicular system [12], MN arrays [13], etc. These nanocarriers possess unique properties that enable them to deform and penetrate deeper layers of the skin, thus facilitating enhanced absorption of low aqueous solubility drugs like MTX.

Microneedle arrays enhance psoriasis treatment by minimally penetrating the stratum corneum with micron-sized needles (typically 50–900 μm), creating temporary microchannels that enable targeted drug delivery to psoriatic lesions in the dermis, when applied topically [14]. This approach bypasses the skin’s lipid barrier, improving bioavailability while avoiding systemic toxicity and first-pass metabolism associated with oral administration [15]. Therefore, developing microchannels enhances efficacy, allowing for both local and systemic effects, thereby minimizing potential adverse effects associated with oral administrations and without the pain associated with traditional hypodermic needles [16].

A transferosome-loaded microneedle array patch overcomes barriers associated with conventional topical formulations through a dual mechanism: MNs physically disrupt the stratum corneum, creating microchannels that bypass the skin’s lipid barrier, while ultra-deformable transferosomes squeeze through these channels and enhance drug solubility and permeation depth [17]. This combination increases transdermal flux by ~3× (to 28.12 µg/cm²/h) compared to conventional gels, enabling sustained delivery and deeper tissue penetration without systemic toxicity [18]. Therefore, the research aims to develop and optimize a transferosome (TF)-loaded microneedle array (TF-MNs), using the design of experiments (DOE) to improve MTX topical distribution for treating psoriasis. Furthermore, the physicochemical characteristics, in vitro skin penetration, and in vivo effectiveness were tested in an animal model to verify its improvement in diffusion [19]. Furthermore, the results were compared with those of commercial MTX formulation. Hence, through improved drug delivery to the afflicted skin with less systemic exposure and side effects, the effective development of TF-MNs for the topical administration of MTX may serve as a useful therapy for psoriasis management.

## 2. Results and Discussion

During the study, TF formulation was prepared by the ethanol injection method. The solvent injection method is widely used due to ease and scalability as reported in earlier literature too [20]. The prepared TFs were further optimized using the design of experiment and characterized for vesicle size, PDI, ZP, and EE (%).

### 2.1. Optimization of Formulation Using 3^2^ Full Factorial Design

A 3^2^ full factorial design was employed to study the influence of various process-independent and dependent variables on the vesicle size and EE. Three independent variables at two levels were used to develop nine batches of TFs loaded with MTX. Table 1 shows the vesicle size in nanometer and EE (%) obtained for each batch fabricated. The results indicated vesicle size and EE (%) ranged between 22 and 169 nm and 13 and 69 nm, respectively. The variability in these results indicates a clear effect of lipid and surfactant concentration during the fabrication of TF. An increase in surfactant concentration generally leads to a decrease in vesicle size. This effect is attributed to the surfactant molecules disrupting lipid bilayers, promoting the formation of smaller vesicles. Moreover, the length of the carbon chain in surfactants also plays a critical role in stabilizing the smaller vesicles due to enhanced hydrophobic interaction within the lipid bilayer, especially in case lipids are longer in chains. In addition, the hydrophilic–lipophilic balance of surfactants affects the vesicles’ size via aggregate formation among vesicles. Additionally, the charge over the vesicles significantly influences the size and stability. Furthermore, the EE depends on several factors, including drug–surfactant interaction, lipid composition, and fabrication techniques [21].

### 2.2. Effect on the Vesicles Size

**Figure 1:**
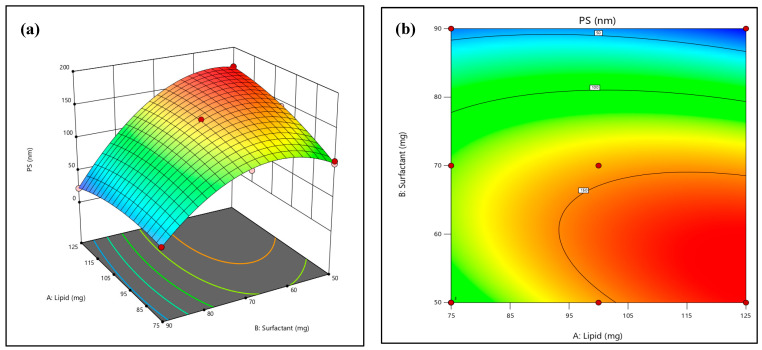
Interaction of lipid and surfactant concentration as illustrated by 3D surface plot of vesicle size (**a**) and 2D surface plot of vesicle size (**b**).

The results indicated that nine compositions exhibited an average vesicle size of 169.4 ± 4.4 (nm). The results suggested that the model best suited to the data for vesicle size was the two-factor interaction (2FI) model. Equation (1) displays the vesicle size equation. A factor’s antagonistic or synergistic effect is demonstrated in the equation by a positive or negative sign before the coefficient. The vesicle size regression coefficient (R^2^) was observed as 0.9959. Furthermore, the effect of designated independent constraints on the vesicle size is demonstrated in 3D plots (Figure 1). The statistically significant correlation between the dependent and independent variables was observed by generating ANOVA data. The 3D surface plot of vesicle size versus factors X1 and X2 is presented in Figure 1a, portraying the decrease in vesicle size with an increase in the amount of lipids. Figure 1b depicts vesicle size versus factors X1 and X2. Hence, increases in solid lipid concentration increased the vesicle size, which is described using Equation (1).

Vesicle size = 143.46 + 12.83 A − 51.82 B − 21.22 AB − 11.40 A^2^ − 49.15 B^2^ (1)

### 2.3. Effect on Entrapment Efficiency

**Figure 2:**
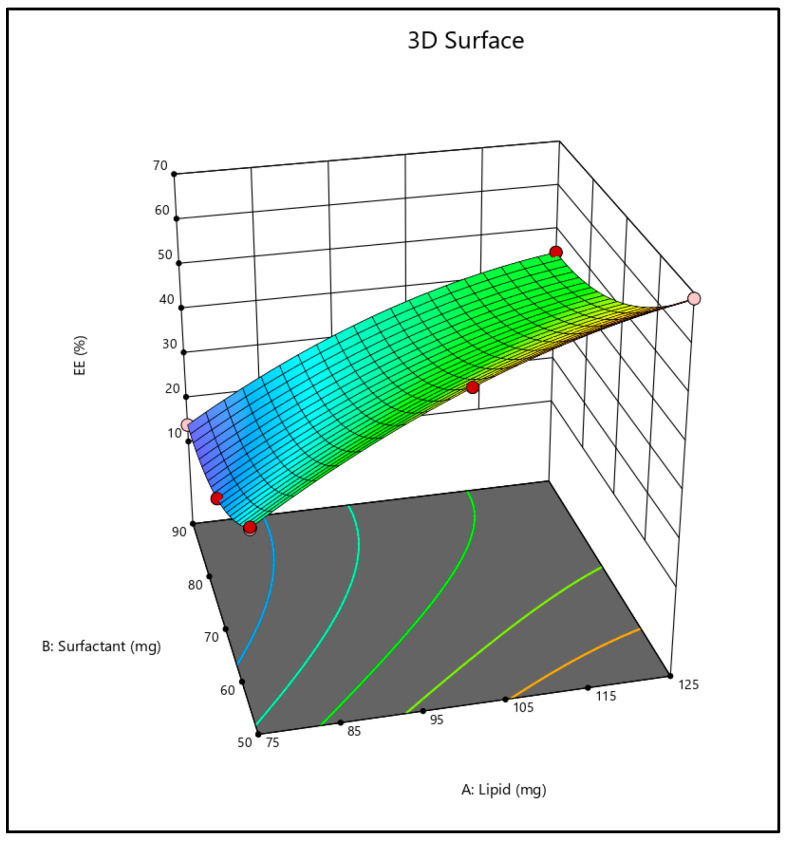
Three-dimensional Surface plot of entrapment efficiency (%).

The EE (%) results suggested that the linear model was best suited to the data. Moreover, the EE (%) plot indicated a regression coefficient (R^2^) value of 0.9998. Figure 2 shows 3D charts illustrating the effect of several independent factors affecting the EE when chosen. The results of ANOVA were exported and evaluated for statistically significant association between the dependent and independent variables. The statistical data suggested that drug EE (%) improved as the quantity of lipid (X1) increased, as observed by the 3D surface plot. Additionally, when X1 was raised from a −1 level to a +1 level, EE (%) at a low and medium level of X2 was considerably (*p* < 0.05) enhanced too.

Entrapment efficiency (%) = +39.52 + 16.62A − 10.92B − 1.01AB − 5.02 A^2^ + 5.96 B^2^ (2)

### 2.4. Optimization of Formulation

The analysis of formulation factors revealed a strong relationship between EE (%), vesicle size distribution, surfactant content, and lipid concentration. A three-dimensional response surface diagram was plotted to highlight the effect of surfactant and lipid interactions that affect vesicle size and EE (%). Based on the data obtained, batch B1 was considered an optimized batch for further study.

### 2.5. Characterization of MTX-Loaded Transferosomes

**Figure 3:**
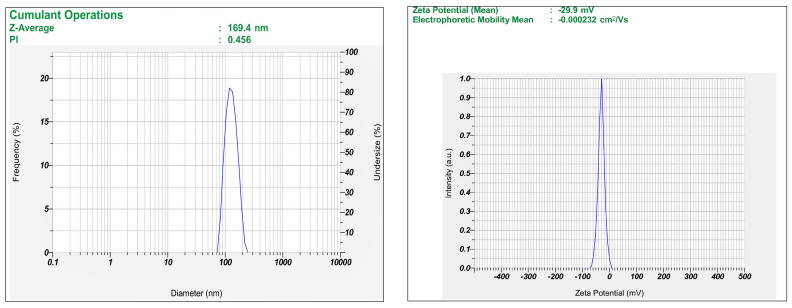
Vesicle size and zeta potential graph of optimized batch (B1).

The developed TFs were evaluated for vesicle size, ZP, and PDI. The vesicle size and PDI of the TF were obtained in a range of 75.9 ± 2.9 nm to 300.7 ± 8.4 nm and 0.300 ± 0.004 to 0.600 ± 0.003, respectively. Due to the existence of phosphatidylcholine (lipid), a high negative ZP of −29.9 ± 0.23 mV was observed. The results suggested that the formulation method and the material quantities are essential and should be adjusted to achieve the desired size distribution. The improved batch (B1) exhibited a PDI of 0.456 ± 0.37 and an ideal vesicle size of 169.4 ± 4.4 nm with a ZP of −29.9 ± 0.23 mV (Figure 3).

#### 2.5.1. Percentage Entrapment Efficiency

The EE (%) of MTX-loaded TF ranged from 13.92 ± 2.58 (%) to 69.22 ± 1.48 (%), suggesting that lipid plays an important role in the EE (%) of the drug. The optimized batch B1 demonstrated an EE of 69.22 ± 1.48%. Thus, with an increase in drug concentration, it was discovered that TF’s EE (%) improved too. This might be due to an increase in the lipid capability to accommodate the higher quantity of the drug [22].

#### 2.5.2. In Vitro Drug Release

**Figure 4:**
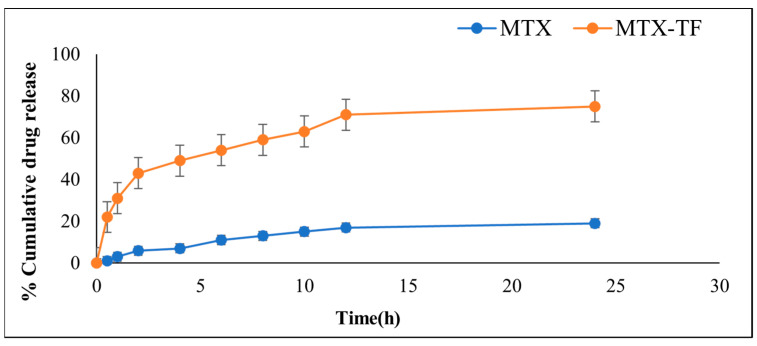
In vitro release study of MTX and MTX-TF.

The results indicated that the initial stage of an in vitro drug release study of TF exhibited burst drug release upon contact with the release medium (Figure 4). Furthermore, a sustained release was observed till 8 h of test duration, indicating that the medication incorporated within the lipid layer’s surface was released. Following this, a steady release was observed for up to 24 h. A formulation showed varying degrees of drug release after 24 h, ranging from 19.9 ± 0.55% to 75.6 ± 0.31%, suggesting that these may have a sustained release pattern. After 24 h, the optimized batch had the maximum drug release at 86.6 ± 0.31%. This result might be due to the drug release profile testing temperature (37.0 ± 0.5 °C) that represents the normal human body temperature, which is below the transition temperature of lipids used in the fabrication of TF and the relative steric hinderance effect of both lipid and surfactant [23].

#### 2.5.3. Transmission Electron Microscopy

**Figure 5:**
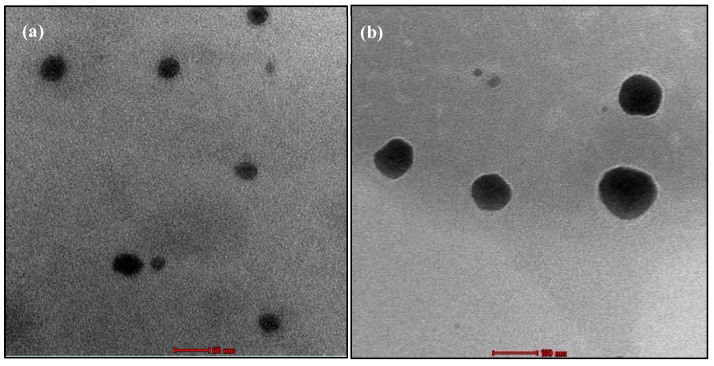
Transmission electron microscopy image of TF at scale bar of 50 nm (**a**) and 100 nm (**b**).

Transmission electron microscopy imaging analysis was utilized to analyze and validate the surface morphology, size, shape, and formation of optimal MTX-loaded TF (Figure 5). The TF loaded with MTX vesicles’ size distribution was uniform and correlated with vesicle size obtained from particle size analyser data. The morphology image indicated a spherical, smooth, and regular surface with no visible aggregation of TF loaded with MTX, suggesting the stable nature of TF.

#### 2.5.4. X-Ray Diffraction

**Figure 6:**
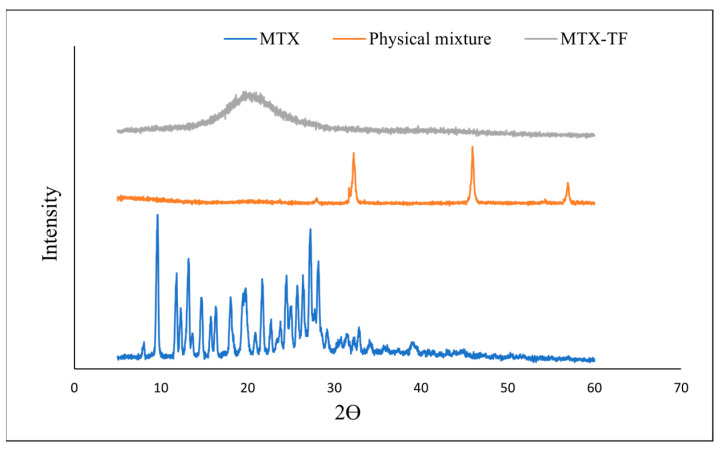
X-ray diffractogram of methotrexate, physical mixture, and optimized TF batch.

X-ray diffraction measurements of MTX, the physical mixture, and the optimized TF formulation (B1) were carried out to understand the drug’s crystal nature (Figure 6). The patterns for MTX showed sharp peaks, indicating the crystalline nature. Although in case of physical mixture of MTX and the auxiliary material, the crystalline peak for MTX was evident; however, peaks for MTX dispersed in the MTX-TF formulation. This might be due to encapsulation of MTX within lipoidal matrix, indicating its presence in an amorphous or molecularly dispersed state. The absence of a peak in the final formulation suggests that the MTX is encapsulated inside the vesicles. In a similar study, curcumin and curcumin metabolites demonstrated spectra in the native form; however, they disappeared on encapsulation within phosphatidyl choline and subsequently reappeared on lyophilisation, suggesting presence of active compound that can show the therapeutic efficacy [12,21]. A study suggested that the therapeutic efficacy is always higher than that of the crystalline form, so the conversion from the crystalline to amorphous form may be beneficial for delivering MTX via skin [24].

#### 2.5.5. Physical Stability Studies

The MTX-loaded TF formulation evaluations recorded during the stability study were 189 ± 29.26 nm with PDI 0.371 ± 0.045 and a zeta potential value of −29.6 ± 0.14 mV. The vesicle size considerably increased in comparison to the original MTX-loaded TF, as has already been noted in related studies, whereas zeta potential and PDI values did not change statistically. In addition, during the 30-day observation period, no discernible flocculation or agglomeration was seen [25]

### 2.6. Fabrication and Morphological Characterization of MTX-MNs and MTX-TF-MNs

A 2:1 ratio of PVA–gelatin combination was prepared to fabricate the MN array patch. A weighed amount of PVA-gelatin mixture in water was heated to 50 °C. After mixing well, the mixture was allowed to cool. PVA–gelatin mixture and TF loaded with MTX were poured into the PDMS molds in predetermined ratios, as mentioned in earlier literature [26].

#### 2.6.1. Characterization of Microneedle Array Patch

**Figure 7:**
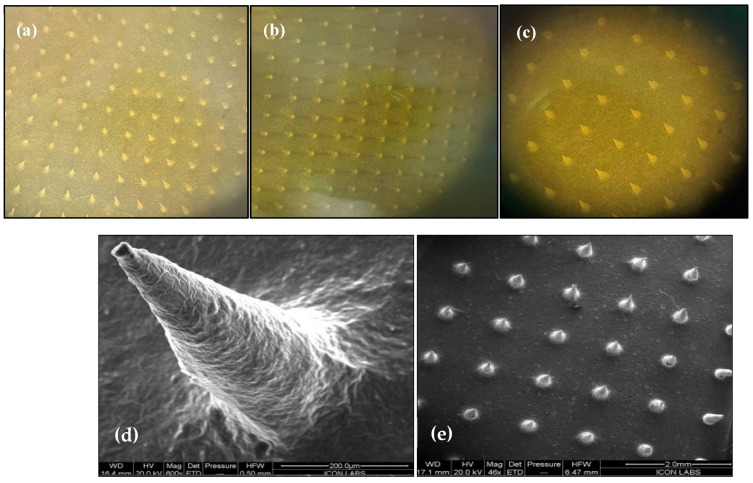
Top view of MTX-loaded TF-MNs at scale bar 2 mm using optical microscopy (**a**–**c**). FESEM image tip of MTX-loaded TF-MN (**d**) at scale bar 200 μm; top view on scale bar 2.0 μm (**e**).

Optical microscopy of MTX-loaded TF-MNs was investigated, and images were presented in Figure 7a–c, while FE-SEM images of the TF-loaded MN array patch loaded with MTX are presented in Figure 7d,e. The image suggested that MNs were shaped with a base diameter of 220.2 µm and an average length of 450.5 µm. The surface of the TF-loaded MNs array patch was flawless, devoid of any fractures or cracks.

#### 2.6.2. Drug Content Determination

Using HPLC, the drug content of MTX-loaded MN and MTX-loaded TF-MN was ascertained. The results showed that the total drug content in MTX-loaded MN, including the baseplate, was 4.5 ± 0.25 mg, whereas the TF-MN loaded with MTX contained 4.8 ± 0.41 mg of medication.

#### 2.6.3. In Vitro Release Study of MTX-MN and MTX-TF-MN

**Figure 8:**
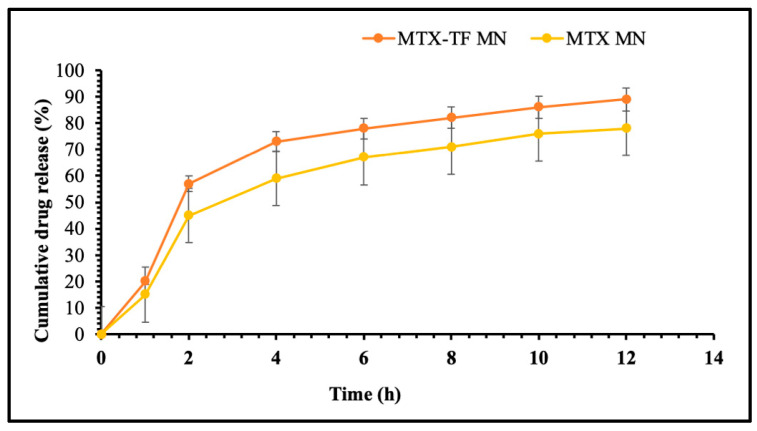
In vitro release study of MTX-MN and MTX-TF-MN.

The MN array patch dissolves in about 30 ± 10 min after application and completely dissolves in about 60 ± 10 min. The release profiles of the MTX-loaded with TF-MN array patch and MTX-loaded MN array patch are displayed in Figure 8. The initial burst release of MTX from the TF-MNs array patch loaded with MTX was observed in 2 h due to the availability of free drugs on the surface of MN and at the base. The further release occurred at 4 h post-MTX coming in contact with the release medium. Moreover, in 12 h, the TF-MN array patch loaded with MTX demonstrated 80.37 ± 5.34% drug release. On the other hand, an initial burst release within 1 h was observed for the MN array patch release profile loaded with MTX. As no obstacle prevented the drug from being released, this could be related to the quantity of the drug that underwent dissolution in the dissolution medium. Within 12 h, about 90 ± 4.17% of the drug release was observed. The higher drug released was reported from the TF-loaded MN array patch containing MTX compared to MTX-loaded MNs due to various barriers such as smaller size, large surface area, and presence of lipid and ethanol, which aid in the higher solubilization capability of TF, which was also reported in earlier studies [27].

The physicochemical properties of the active compounds as well as polymeric materials influence the release of active compounds from the formulation and, thus, modify the release kinetics accordingly. The mathematical drug release models with major application and best describing drug release phenomena are, the Higuchi, zero order, first order, Korsmeyer–Peppas, and Hixon Crowell models [28]. Thus, these release kinetic models were applied and the results of in vitro release kinetic modeling showed that release of drug followed first order of release with an r^2^ of 0.9228. The Higuchi equation suggests drug release by diffusion, whereas the Korsmeyer–Peppas power law equation defines the type of diffusion, while Hixon Crowell model suggest erosion mechanism of polymeric matrix allowing release of drug. The results suggested that the release of MTX was dependent of concertation and governed by diffusion.

#### 2.6.4. Ex Vivo Permeation Study

**Figure 9:**
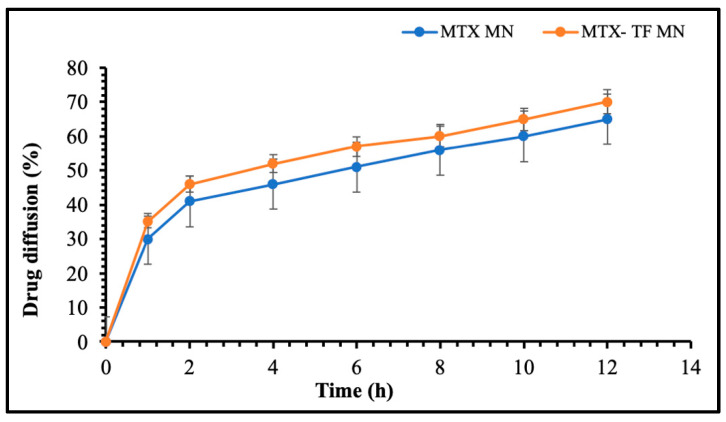
Ex vivo permeation study MTX-MN and MTX-TF-MN.

The ex vivo result is presented in Figure 9; the result demonstrated that compared to the MTX-loaded MN array patch, the TF-MN array patch showed a 1.13-fold improvement in drug diffusion. The lipophilic nature and presence of ethanol, which acts as a permeation enhancer as reported in the literature of the TFs in the MN array patch, is responsible for the drug’s increased diffusion [6]. Since MTX is a hydrophilic medication, it dissolves easily in lipid-based TFs. This enhanced permeability, together with TFs’ interaction with the lipid barrier of the skin, suggested that MTX diffusion may improve and allow ease in passage through the stratum corneum, the outermost layer of the skin. Additionally, the prolonged-release profile and environmental degradation protection offered by TFs also provide a steady and regulated supply of TF throughout time, which accounts for the drug’s reported improved penetration and uptake.

#### 2.6.5. In Vivo Pharmacokinetic Assessment

**Figure 10:**
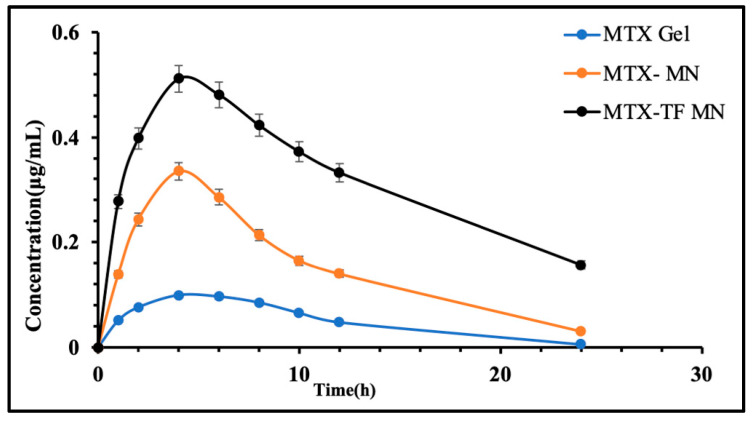
In vivo pharmacokinetic study in plasma.

To compare the quantity of MTX in plasma after delivery, an in vivo pharmacokinetic study was tested on the MTX-loaded marketed gel, MTX-loaded MN array patch, and MTX-loaded TF-MN array patch. Figure 10 displays the drug concentration versus time curve of plasma for the three study groups. Table 2 shows the MTX AUC, C_max_, T_max_, and t_1/2_ levels in plasma following the MTX-loaded MN array and MTX-loaded TF-MN array patch application. The results suggested that the TF-MN array patch loaded with MTX accumulated twice as much MTX, compared to MTX-MN. This result implies that the TF formulation enhances the medication transport to the plasma. Additionally, the TF-MN array patch loaded with MTX has a longer half-life (t_1/2_) in plasma, suggesting a sustained release pattern that may improve the therapeutic efficacy and duration of action. A study on MTX-incorporated niosomes tested for in vivo deposition with claiming reduced systemic cytotoxic effect showed a high AUC_0–10_ of 1.15 mg/h/cm^2^ [29], while another study on liposome vesicles that incorporated MTX showed a high transdermal flux with no signs of irritation or toxicity [30], suggesting that prepared MNs fortified with MTX incorporated within TF can be used effectively to treat psoriasis in an improved form. These results suggest a beneficial approach for the treatment of diseases like psoriasis.

#### 2.6.6. Histopathology Study

**Figure 11:**
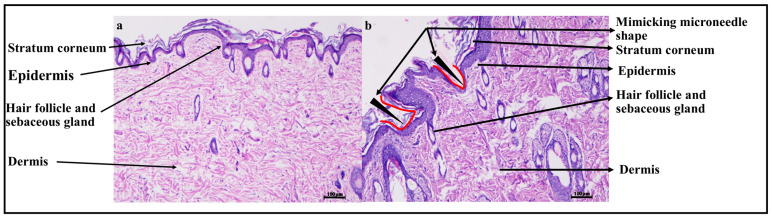
Microscopic images of dorsal skin tissue obtained for histopathology study. Normal skin (**a**), skin after insertion of MTX-loaded TF-MN (**b**). The red line showing a rough structure formation after insertion of microneedle to the skin.

The histopathology investigations aimed to determine if the MN could effectively penetrate the skin layers using skin tissue from male Wistar rats. In ex vivo tests, TF-MN loaded with MTX was used to puncture the isolated dorsal skin of Wister rats for 10 min, and then it was withdrawn. Following tissue isolation, it was preserved in 10% formalin. H&E was used to stain the slides after preparing them according to the standard procedure. Furthermore, the slides were investigated using a microscope. Figure 11a discusses the results for normal skin, suggesting that the dermis and epidermis layers were intact and normal, and the subcutaneous tissue’s histomorphology included a normal hair follicle and sebaceous glands. However, Figure 11b demonstrates that MNs punctured the skin about a depth, penetrated the stratum corneum forming a cone-like hole, and delivered drug directly beneath the epidermis without disturbing the normal architecture of the epidermis, hair follicles, and sebaceous glands. The TF-loaded MTX is designed to release the drug below the epidermal layer, where it predominantly accumulates. Subsequently, the drug penetrates from the epidermis into the dermis. However, systemic absorption of MTX via the capillary network in the dermal layer is minimal, primarily due to the hydrophilic nature of the drug, as confirmed by in vivo pharmacokinetic studies using PK solver software (Version 2.0). Another critical factor contributing to the reduced systemic absorption is the length of the MNs, measured at approximately 280 µm. Considering that the combined thickness of the epidermis and dermis in adult rat skin is approximately 300 µm, the ideal MN length targets the epidermis and upper dermis while avoiding deeper penetration into regions containing blood capillaries. In this study, the designed microneedle length ensures precise delivery of the drug within the epidermis and upper dermis, effectively preventing systemic absorption. This targeted approach minimizes the risk of systemic side effects associated with MTX administration [31].

## 3. Materials and Methods

### 3.1. Materials

Methotrexate (Mw: 454.4 g/moL of Purity: 98%) was received as a gift sample from Khandelwal Laboratories Pvt. Limited, Mumbai, India and Chemspace Pharmaceuticals Private Limited, Mumbai, India. Leciva S90 was obtained as a gift excipient from VAV Lipids, Mumbai, India. Polyvinyl alcohol (Mw: 31,000–50,000 g/moL) and gelatin A (Mw: 50,000 to 100,000 g/moL (kDa); gelatin from porcine skin) were procured from Sigma Aldrich, St. Louis, MO, USA. The formulation and buffers were prepared using Milli-Q water (PURIST Ultrapure Water System Instrument, RephiLe Bioscience, Boston, MA, USA). The solvent utilized was of HPLC grade, while all other compounds, components, and reagents were of analytical grade. The MTX-marketed gel (Folitrax LP Methotrexate Gel, manufactured by IPCA Laboratories Ltd., Mumbai, India) was purchased for an in vivo investigation.

### 3.2. Method

#### 3.2.1. Preparation and Characterization of MTX-Loaded Transferosomes

**Figure 12:**
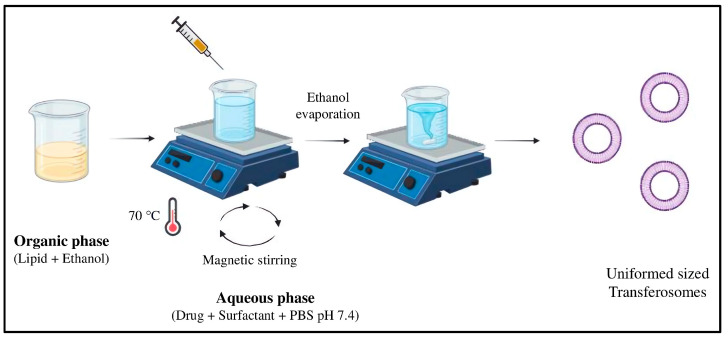
Method of preparation of transferosomes by ethanol injection method.

As reported previously, MTX-loaded TF batches (a total of nine) were formulated using the ethanol injection technique [32]. Table 3 shows formulation parameters such as lipid concentration and surfactant concentration, which were explored to optimize the fabrication of TF. Briefly, 10 mg of MTX and Tween 80 were admixed in 10 mL of phosphate buffer (pH 7.4) to produce the aqueous phase. Leciva S90 was dissolved in 4 mL of anhydrous ethanol while being stirred magnetically at 600 RPM (Magnetic stirrer, Remi 1 MLH, India) to develop a transparent solution that formed the organic phase. Subsequently, both solutions were heated to 70 °C and stirred magnetically at 600 RPM. After that, the aqueous solution was continuously stirred for 20 min while a dropwise injection of the ethanolic phospholipid solution was added. Figure 12 shows the method of preparation of TF by the ethanol injection method.

#### 3.2.2. Experiment Design for Optimization

Design-Expert (Design Expert Software, version 13.0.5, Stat-Ease, Minneapolis, MN, USA) employing 2-factor, 3-level, 3^2^ full factorial designs suggested 9 runs to maximize the MTX loading within TF. Lipid concentration (X1) and surfactant concentration (X2) were chosen as independent variables. Vesicle size (Y1) and EE (Y2) were taken as dependent variables. Table 4 shows that each independent variable varies in triplicate, with higher, medium, and lower values.

Equation (3) was produced by analyzing the data acquired for nine batches using the Design Expert (version 13, Stat ease).

Y= β0 + β1X1 + β2X2 + β3X3 + β12X1X2 + β13X1X3 + β23X2X3 + β123X1X2X3(3)

where X1 and X2 are the effect factors at each level (independent variables); β0 is the intercept; R1–R33 are the regression coefficients of the respective variables and their interactional terms calculated by experimental data, whereas R is the measured response (dependent variable) about each level of independent variables of the study design.

#### 3.2.3. Optimization of the Formulation

Combining data from several dependent variables and statistical analysis, an optimum formulation with projected values for formulation components opted such lipid and surfactant content. Vesicle size, EE, and drug release percentage were among the dependent variables whose outcomes were also predicted by the procedure. The optimal formulation formula was obtained and validated the model using all the data and examined for vesicle size, polydispersity index (PDI), zeta potential (ZP), EE, and release kinetics.

#### 3.2.4. Size, Polydispersity Index, and Zeta Potential Analysis of Fabricated Vesicles

The vesicle size distribution, PDI, and ZP were ascertained using a size analyzer (HORIBA Scientific, Nano series SZ-100, Kyoto, Japan) as reported previously [33]. In brief, each formulation sample of 1 mL was diluted with 9 mL of MiliQ water (PURIST Ultrapure Water System Instrument, RephiLe Bioscience, Boston, MA, USA), and three measurements were made at room temperature at 90°.

#### 3.2.5. HPLC Analysis of Methotrexate

The quantitative chromatography profiling of MTX was performed using JASCO high-performance liquid chromatography (HPLC) (JASCO, Tokyo, Japan), and results were recorded using Borwin (version 2.0) software-operated HPLC as reported previously [34]. Briefly, separations via reversed-phase chromatography were accomplished using the Thermo C-18 hypersil GOLD column. Phosphate buffer (pH 7.4): acetonitrile (92:8, *v*/*v*) at a flow rate of 0.8 mL/min was used as the mobile phase to separate the components. The eluent was quantified using a detector at a wavelength of 303 nm, sample injection volume was 20 μL, retention duration was 10.6 min, and experiments were tested at a column temperature of 25 °C. The LOD and LOQ of the developed method obtained as 0.6544 μg/mL and 1.9830 μg/mL, respectively, were used for quantitative chromatography profiling of MTX.

#### 3.2.6. Entrapment Efficiency

The quantity of free drug in 1 mL of TF dispersion loaded with MTX was measured using the centrifugation (Beckman Coulter, Allegra 64R Benchtop Centrifuge, Brea, CA, USA) technique to calculate the EE (%) as reported [35]. In brief, a 1 mL produced mixture was centrifuged using colling centrifuge (Eppendorf centrifuge, 5810 R, Hamburg, Germany) for 30 min at 14,000 RPM at 4 °C. The resulting supernatant was subjected to an HPLC analysis to determine the free MTX concentration. The EE (%) was determined using the following equation.

(4) $$Entrapment\;efficiency(\%)=\frac{Total\;amount\;of\;drug\;added-Avilabel\;free\;drug}{Total\;amount\;of\;drug\;added}\times 100$$

#### 3.2.7. In Vitro Drug Release

As reported previously, the dialysis bag technique was used to quantify in vitro drug release [36]. Briefly, post immersion in water for an overnight activated the dialysis bag (12–14 KDa molecular weight cut-off, HiMedia Laboratories Pvt. Ltd., Thane, India). An in vitro release evaluation of a free drug (MTX) and MTX-loaded TF was conducted. A volume of 1 mL of free drug (MTX) and MTX-loaded TF dispersion was placed into the dialysis bag and sealed from both ends. Using a constant 50 RPM stirring speed, the dialysis bag was dipped in a beaker filled with 100 cc of pH 7.4 phosphate buffer as a dissolving media. At specified intervals of 1, 2, 4, 6, 8, 10, 12, and 24 h, aliquots of samples were removed. After removing the aliquots, the same volume of the new dissolving medium was added to maintain the sink condition.

#### 3.2.8. Morphological Characterization of Transferosomes

Transmission electron microscope (TEM) (JEOL, JEM-2100F, Tokyo, Japan) with a super twin lens was used to study the morphology of TF as reported [37]. In brief, a drop of MTX-loaded TF dispersion was placed on a carbon-coated copper grid and stained with 1% sodium phospho-tungstic solution. Later, the excess fluid was removed using absorbent filter paper, and the sample was allowed to dry for 15 min at room temperature before being observed under TEM (Voltage: 200 kV and Resolution: 40,000×) to obtain images and a randomly selected area electron diffraction (SAED).

#### 3.2.9. X-Ray Diffraction

To evaluate the crystal nature of the MTX, mannitol, and MTX-TF batch, an X-ray diffractometer (XRD; Diffractometer, Rigaku Miniflex II, Tokyo, Japan) was used to capture the XRD pattern using a method as reported earlier [38]. A Lindeman capillary was used to collect the powder sample placed on the diffractometer pan. The fixed 90 mm distance between the sample and detector was accompanied by angles of 2θ, ∞, and y of 28°, 14°, 180°, and −90°, respectively. MTX, mannitol, and MTX-TF were plotted with background correction using a PXRD graph.

#### 3.2.10. Physical Stability Studies

The objective of this test was to find out if the produced vesicles leaked any drugs while being stored. The produced vesicles were kept for 30 days at 4 °C in refrigeration conditions in clear vials with plastic caps. The presence of flocculates and agglomerates was monitored daily. The MTX-loaded TFs were evaluated based on vesicle size, PDI, and zeta potential value, as described in Section 3.2.4. The results were compared to the original MTX-loaded TF.

### 3.3. Fabrication of Microneedle Array Patch

**Figure 13:**
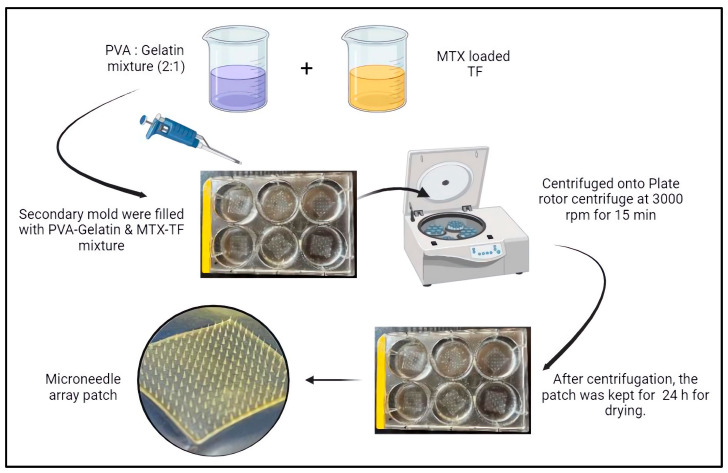
Illustration showing fabrication process microneedle array patch loaded with MTX-TF.

A master mold containing metallic MNs was constructed utilizing the outsourced facility. Polydimethylsiloxane (PDMS) compound, a blend of elastomer and curing agent in a 10:1 ratio, was mixed and degassed. It was placed in the master mold to develop a secondary mold. The compound was then heated in an oven at 90 °C for 50 min. Upon separating the cured material from the master mold, the MN array patch production process may commence. MTX-loaded TF and a PVA-gelatin combination were added to the PDMS molds in predetermined quantities. Later, the molds were then placed onto the plate rotor of the centrifuge (Eppendorf centrifuge, 5810 R, Hamburg, Germany) and spun for 10 min at 3000 RPM. Following centrifugation, the mold was allowed to dry for 24 h at room temperature. Then, the MNs were removed from the mold post drying. Similarly, MN of alone MTX was prepared to compare the pharmacokinetic parameters. Figure 13 provides the diagrammatic illustration of the fabrication of the MTX-TF loaded MN array patch.

### 3.4. Morphological Characterization of MTX-TF-Loaded MNs

#### 3.4.1. Optical Microscopy and Field Emission Scanning Electron Microscopy

Optical microscopic (Microscope, Olympus SZX16, Tokyo, Japan) photographs of MN array patches were imaged from various directions and different viewpoints to analyze the shape, sizes, and arrangements of the needles in the array. Furthermore, the morphology of needles produced for the MTX-loaded TF MNs patch was studied using field emission scanning electron microscopy (FE-SEM; JEOL, JSM 7600F, Tokyo, Japan).

#### 3.4.2. Determination of MTX Content in MTX-MNs and MTX-TF-MNs

The presence of MTX within MTX-MNs and MTX-TF-MNs was quantified using a modified method as reported [39]. In brief, the MNs patch was dispersed into a glass vial with 10 mL of water, sonicated for 15 min and diluted with 10 mL of acetonitrile. Subsequently, further sonicated for an additional 15 min to determine the drug content of MTX-loaded MN and MTX-loaded TF-MN. The drug was then left dissolved while 200 μL was placed into 2 mL Eppendorf tubes and combined with 800 μL acetonitrile to precipitate PVA polymer. Later, the supernatant was collected for HPLC examination post centrifugation (Eppendorf centrifuge, 5810 R, Hamburg, Germany) of this dispersion for 10 min at 12,000 RPM. The experimental readings of each experiment were taken in triplicate.

#### 3.4.3. In Vitro Release Assay for MTX-MN and MTXTF-MN

The in vitro release study of alone MTX-MN and MTX-TF-MN loaded with a pure medication and the other with MTX-TF, respectively, was tested using a modified method with USP type II dissolution equipment (Electrolab, TDT-08L, Navi Mumbai, India) as reported [10]. In brief, a single MN array patch was suspended for the release at a speed of 50 RPM in 900 mL of phosphate buffer pH 7.4 (release medium) at 37 ± 0.5 °C. To maintain sink condition, samples (5 mL) were withdrawn and replaced at different intervals (1, 2, 4, 6, 8, 10, and 12 h) with an equivalent fresh medium volume to maintain sink condition. The quantity of drug release was measured using HPLC analysis as the method indicated in Section 3.2.5. Furthermore, the results of in vitro release was fitted to mathematical models such as zero order, first order, Higuchi, Hixon Crowell, and Korsmeyer–Peppas to understand the release profiles and effect of incorporating the drug within lipoidal TF and subsequently to MNs.

#### 3.4.4. Ex Vivo Permeation

A rat dorsal skin was used to investigate the permeation of the drug utilizing the Franz diffusion cell. The study was conducted on the dorsal skin of male Wistar rats. Initially, 6 male rats of 120–150 gm were split into two groups. After acclimatization, the rats were sacrificed by CO_2_ overdose, and the dorsal skin, in a circular shape, 5 cm in diameter, of all the rats was trimmed gently to remove the furs without disturbing the skin. Later, the shaved area was gently sponged with lukewarm water to remove any leftover hairs. Finally, 5 cm diameter ring of full-thickness skin was excised and again thoroughly washed under running water. The excised rat dorsal skin was used in the permeation investigation utilizing the Franz diffusion cell. Phosphate buffer pH 7.4 was utilized as the diffusion medium in the receptor chamber. The skin was carefully placed onto the receptor compartment, to maintain the continuous contact of stratum corneum with the donor compartment. The assembly was maintained at 37 ± 0.5 °C using a magnetic stirrer (Remi, R24, India) running at 50 RPM (a magnetic bead was added to the receptor compartment for continuous stirring). The pure MTX-loaded MNs and MTX-TF-loaded MN array patches were applied to the skin. To maintain sink condition, samples (2 mL) were withdrawn and replaced at different intervals (1, 2, 4, 6, 8, 10, 12, and 24 h) with an equivalent fresh medium volume to maintain sink condition. The quantity of drug diffused was measured with an HPLC analysis as the method indicated in Section 3.2.5.

#### 3.4.5. In Vivo Pharmacokinetic Assessment

The ex vivo study findings showed that MTX-TF-MN might offer a prolonged and effective drug release by delivering MTX-TF into the skin. However, an in vivo environment is required to verify the results obtained. To further understand the effectiveness of the fabricated MTX through either loaded in TF or in pure for fortifies within MNs array patch drug delivery system, an in vivo investigation was conducted and compared with MTX-marketed gel (Folitrax LP Methotrexate Gel, manufactured by IPCA Laboratories Ltd., Mumbai, India). A total of 24 male Wistar rats weighing between 180 and 210 g was used. The animal experiments were prior approved by the Government of India’s Committee for the Control and Supervision of Experiments on Animals (CCSEA) and Institutional Animal Ethics Committee (IAEC) with approval No. PCP/IAEC/2024/2-23. To quantify the pharmacokinetics of MTX, rats were split into 3 groups (*n* = 8) and treated with MTX-TF-MNs, MTX gel, and MTX-MN. Rats were housed at 37 °C ± 1 °C with a 12 h light–dark cycle and relative humidity of 30 ± 10% for re-habitation. Normal lab food and water were provided to each rat. Briefly, the animals were anesthetized using 2.5% isoflurane, and the dorsal side of the animals was trimmed before the application of MTX-TF-MNs, MTX gel, or MTX-MN patches. After hair removal, MTX-loaded pure MNs patch, MTX-loaded TF-MNs patch, and MTX-marketed gel were applied over test rats on the shaved dorsal area. To access the MTX plasma levels, periodic blood samples were collected from all the rats in different groups (Groups I, II, III) by retro-orbital puncture at (1, 2, 4, 6, 8, 10, 12, and 24 h). Around 0.15–0.175 mL of blood was withdrawn through retro-orbital puncture under light anesthesia. The blood samples were centrifuged (15,000 RPM, 10 min, 4 °C) to separate the plasma. To recover MTX from the plasma, the plasma was added to an Eppendorf tube with methanol for protein precipitation. The samples were vortexed for 1 min and, sequentially, centrifuged at 15,000 RPM for 10 min at 4 °C. The supernatant was collected and analyzed by using the HPLC analysis as the method described in Section 3.2.5. All the experiments were conducted in triplicate, and results were expressed as mean ± SEM.

#### 3.4.6. Histopathology Study

A histological examination was performed to find out how well the MNs’ array patch penetrated the layers of the skin. Male Wister rats of 120 to 150 gm were split up into two groups, each comprising three animals. All animals were anesthetized using an isoflurane vaporizer on the dorsal side of 5 cm diameter circle area (78.5 cm^2^) was trimmed to remove the furs cleaned with lukewarm water and allowed to dry. Group 1 animals were treated with a placebo patch whereas for group 2 rats, TF-loaded MNs array patch was applied. Ten minutes after the application, the patches were removed from all the rats, and the skin was excised (where the patches were applied) after the animals were sacrificed. The excised skin was gently washed under running normal saline to remove the surface debris. The collected skin tissues were placed on a cryostat specimen holder and embedded in a suitable cryoprotectant medium. The chunk was rapidly shifted to the cryostat chamber to freeze at a temperature of −20 °C to −30 °C. The frozen sample was sectioned into thin slices (5–10 μm) using the cryostat. Immediately, the sectioned samples were stained with hematoxylin and eosin (H&E). The stained sections were observed under a light microscope at 100× magnification to evaluate the depth of MN penetration, the structural integrity of the epidermis, dermis, and underlying layers and any tissue disruption or inflammation caused by the MN patch, and the outcome is compared among the two groups.

### 3.5. Statistical Analysis

All the analysis of samples was performed in triplicate, and results were noted as mean ± SD and mean ± SEM. GraphPad Prism software was used for statistical analysis of all the results obtained (Version 8.0, GraphPad Software Inc., La Jolla, CA, USA).

## 4. Conclusions

In conclusion, this research study demonstrates the potential of the MTX-loaded MN array patch as a highly effective method for topical MTX delivery in the treatment of psoriasis, increasing MTX release. The developed formulation also showed 2 to 3-fold higher drug accumulation and reduction in systemic exposure compared to conventional formulations. It highlights the system’s ability to address key challenges in psoriasis management, such as minimizing side effects while maintaining efficacy. Histological analysis further confirms the ability of the fine needle patch to effectively avoid the obstruction of the epidermis. These findings reinforce the promise of transferosome-loaded microneedles as an innovative, safe, and effective platform for the local treatment of psoriasis. Future research and clinical trials are warranted to examine these results in human populations. and optimize this technology for clinical use. Furthermore, investigating the use of this technology for additional dermatological disorders like vitiligo or eczema may broaden its therapeutic potential. Investigating combination therapy, in which methotrexate is used in conjunction with additional topical medications, may improve treatment results even further. Finally, to confirm these results and open the door for therapeutic use, in vivo research in bigger animal models or human clinical trials is crucial. Researchers may fully realize the promise of microneedle-based delivery devices for dermatological therapies by exploring these possibilities.

## Figures and Tables

**Table 1:** Responses obtained from studied parameters for experimental batches.

Batch	Y1 Vesicle Size (nm)	Y2 Entrapment Efficiency (%)
B1	169.4 ± 4.4	69.22 ± 1.48
B2	22.1 ± 3.9	45.14 ± 0.74
B3	40.1 ± 4.9	13.92 ± 2.58
B4	100.4± 0.1	33.85 ± 1.86
B5	116.8 ± 3.2	17.91 ± 0.37
B6	99.8 ± 2.1	33.94 ± 0.61
B7	145.9± 0.5	39.52 ± 1.34
B8	103.7 ± 1.6	33.47 ± 3.48
B9	143.7 ± 1.4	56.42 ± 0.51

Results expressed as Mean ± SD, *n* = 3.

**Table 2:** Pharmacokinetic parameters.

Parameters	Plasma		
	MTX Marketed Gel	MTX-MN	MTX-TF-MN
T_max_ (h)	2.5 ± 0.0	3.1 ± 0.15	3.4 ± 0.17
C_max_ (μg/mL)	0.25 ± 0.16	0.47 ± 0.27	0.50 ± 0.41
AUC_0–t_ (μg/mL·h)	2.25 ± 0.51	4.94 ± 0.27	7.68 ± 0.24
AUC_0–∞_ (μg/mL·h)	2.27 ± 0.17	5.15 ± 0.21	10.26 ± 0.11

All time points were performed in triplicates (Mean ± SD).

**Table 3:** Independent and dependent variables for optimization of TF using DOE.

**Independent Variables**	**Levels**		
	**Low (−1)**	**Medium (0)**	**High (+1)**
X1 lipid concentration (mg/mL)	75	100	125
X2 surfactant concentration (mg/mL)	50	70	90
**Dependent Variables**			
Y1	Vesicle size (nm)		Lower
Y2	EE (%)		Highest

**Table 4:** Composition of MTX-loaded TF batches as per Design-Expert 13.

Batch	LS-90 (X1)	Tw-80 (X2)
B1	125	50
B2	125	90
B3	75	90
B4	75	50
B5	75	70
B6	75	50
B7	100	70
B8	75	50
B9	100	50

## Data Availability

Data are contained in the paper.
